# Supplementary figures and images for: Genetic and environmental drivers of large-scale epigenetic variation in Thlaspi arvense
Source: PLoS Genet. 2022 Oct 12;18(10):e1010452. doi: 10.1371/journal.pgen.1010452 (PMC9591053; doi:10.1371/journal.pgen.1010452)

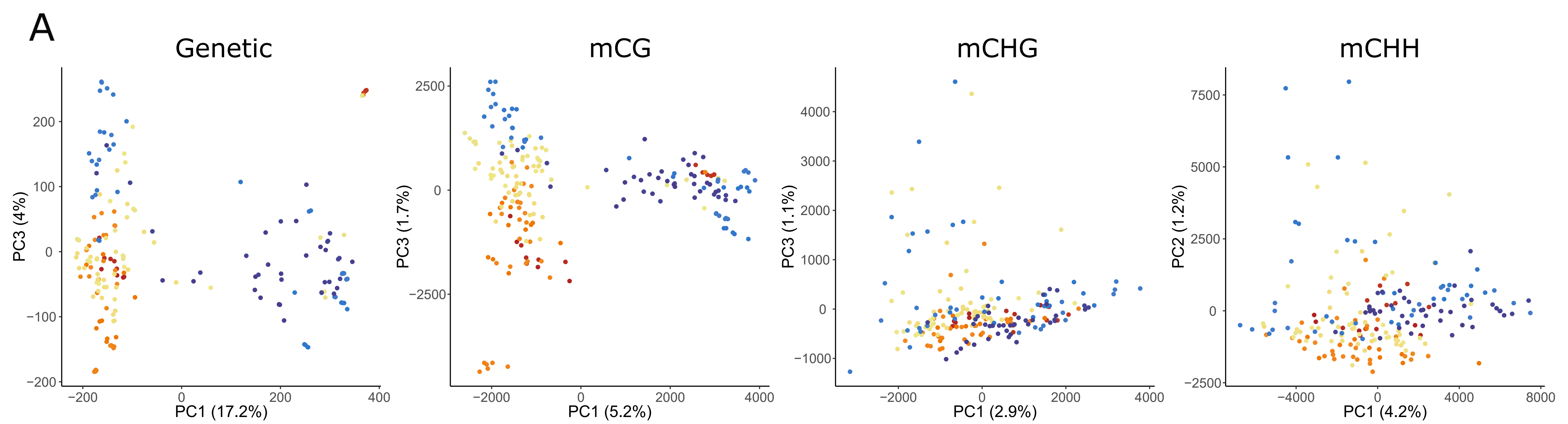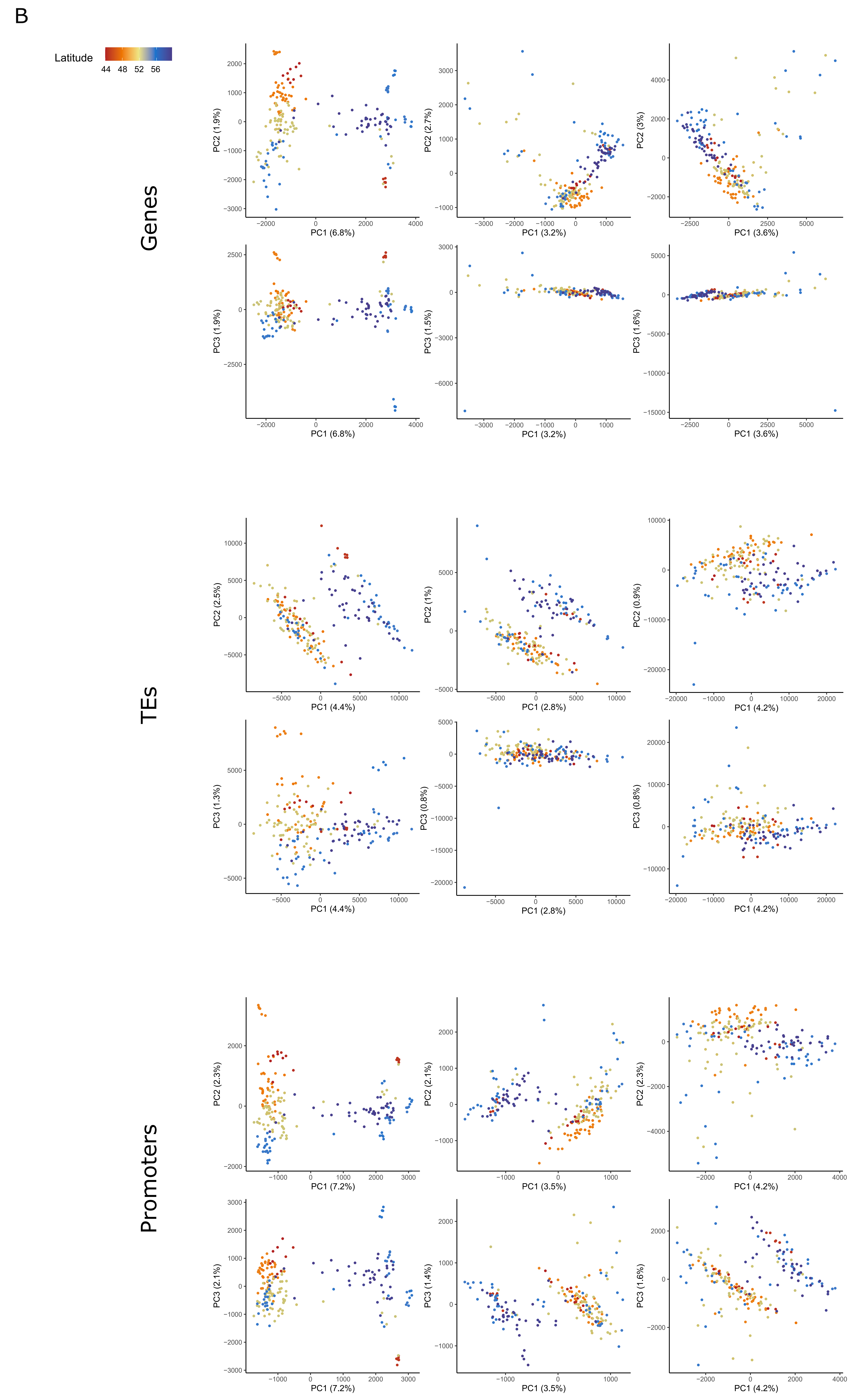

Supplement: S1 Fig — (A) Complement to Fig 1B with latitude-coloured PCA plots for the missing PC. (B) latitude-coloured PCA plots based on methylation of specific genomic features (genes, TEs and promoters). (PDF) [file pgen.1010452.s001.pdf]

C

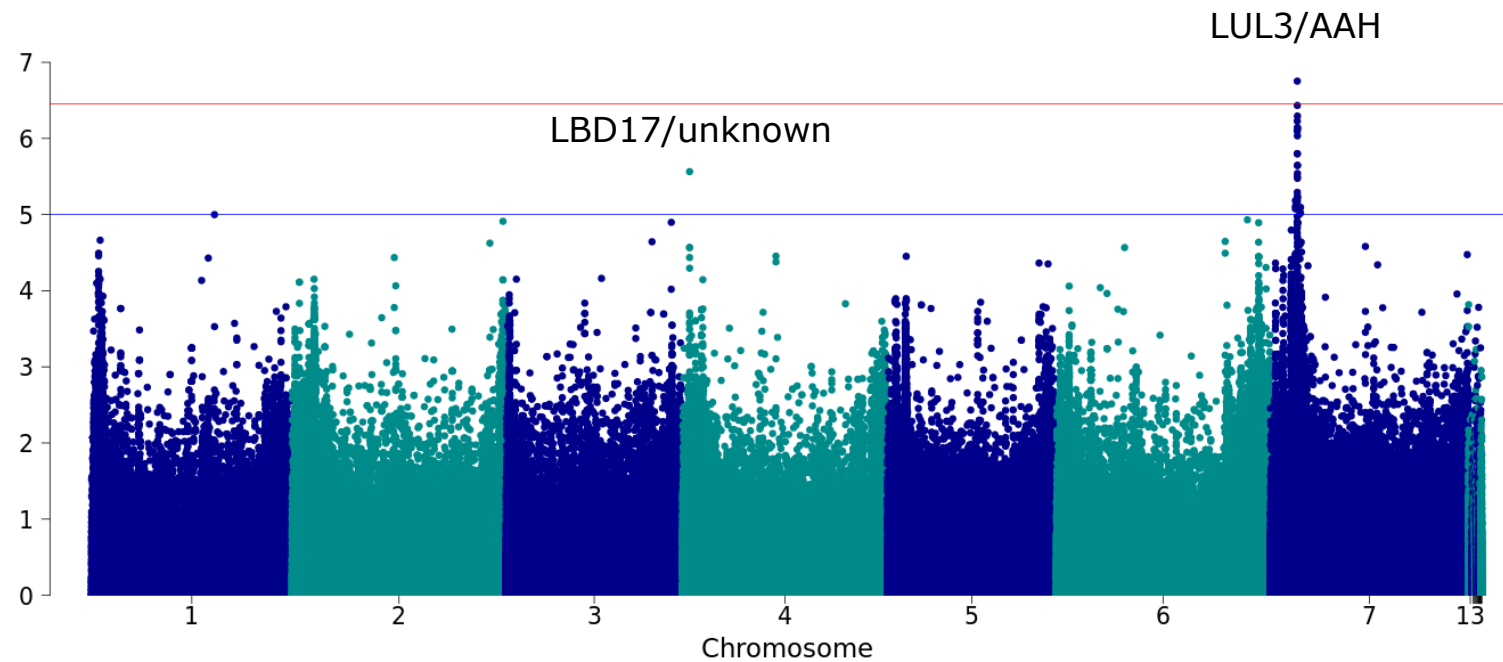

## Enrichment

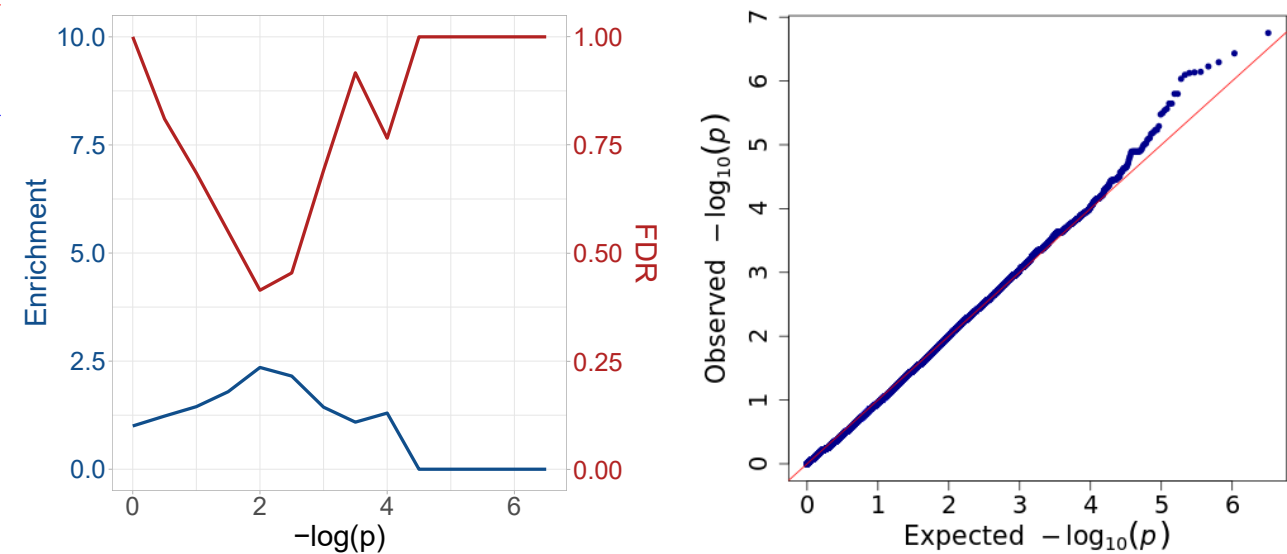

Supplement: S2 Fig — (A) Venn diagram of the number of genes methylated in each context in at least 70% of the lines, which were also used for the GO enrichment. Genes methylated only in CG are labelled as “gbM”, genes methylated in either CHG or CHH as “TE-like” [12]. (B) GO enrichment analysis of methylated genes corresponding to (A). Only significant results for GO terms with minimum gene count of four are reported. GO categories are: Biological Process (BP), Cellular Component (CC) and Molecular Function (MF). (C) GWA for number of gbM genes, including Manhattan plot, enrichment of a-priori candidates and qqplot. (PDF) [file pgen.1010452.s002.pdf]

GO term

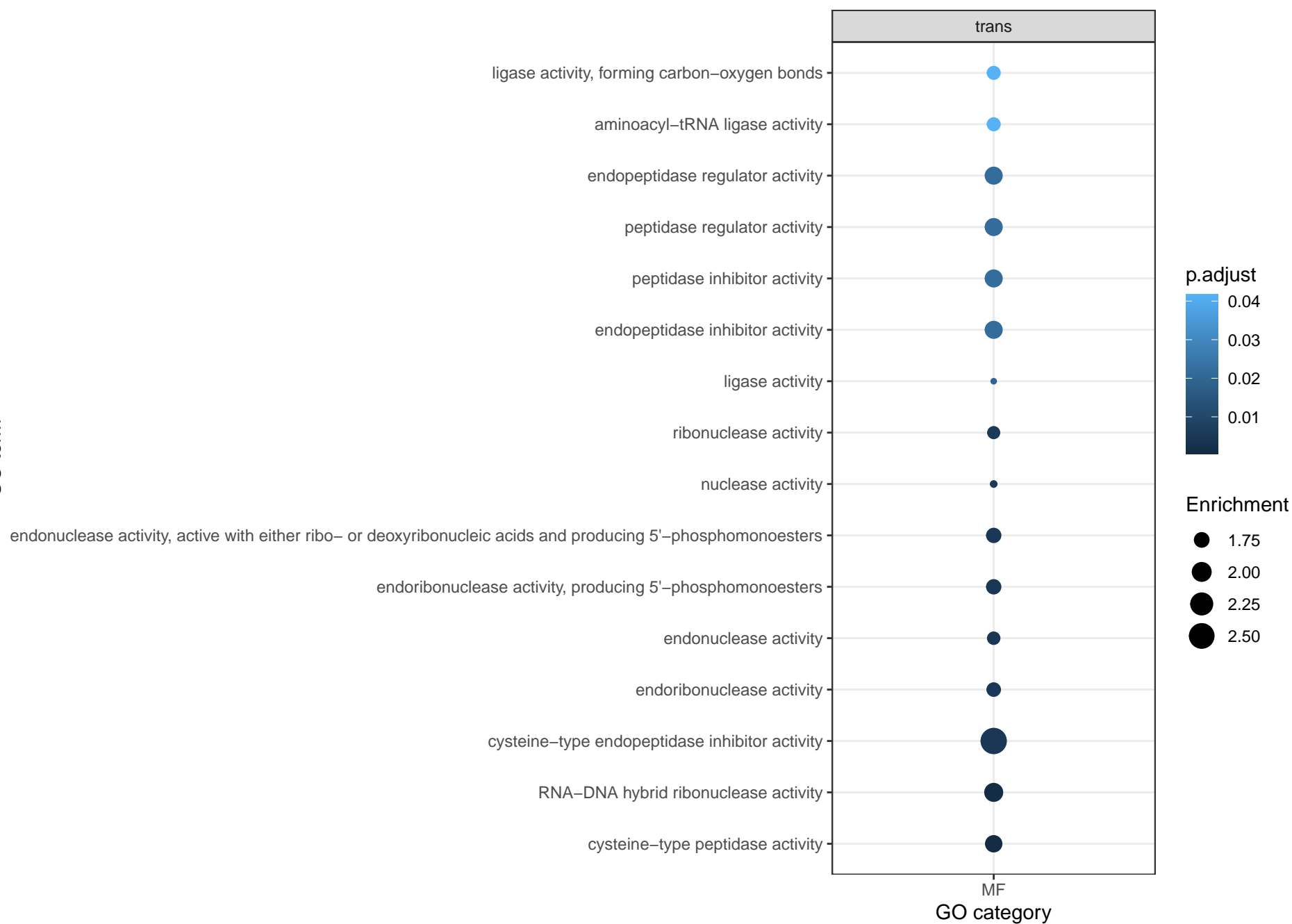

Supplement: S4 Fig — Genes neighbouring (2kb max) cis, trans and env-DMRs were used for individual GO term enrichment analysis, but only the trans-DMRs gene set was enriched for any significant term. (PDF) [file pgen.1010452.s004.pdf]
