# Supplementary material for: Genetic and environmental drivers of large-scale epigenetic variation in Thlaspi arvense
Source: PLoS Genet. 2022 Oct 12;18(10):e1010452. doi: 10.1371/journal.pgen.1010452 (PMC9591053; doi:10.1371/journal.pgen.1010452)
Supplement: S3 Fig — Manhattan plots, enrichment of a priori candidate variants and QQplots for all mean methylation phenotypes. more5met: mean methylation of genes with methylation > 5% across all lines. The genome-wide significance (horizontal red lines), was calculated based on unlinked variants as in Sobota et al. (2015) [49], the suggestive-line (blue) corresponds to–log(p) = 5. Top variants are labelled with the neighbouring genes potentially affecting methylation. (PDF) [file pgen.1010452.s003.pdf]

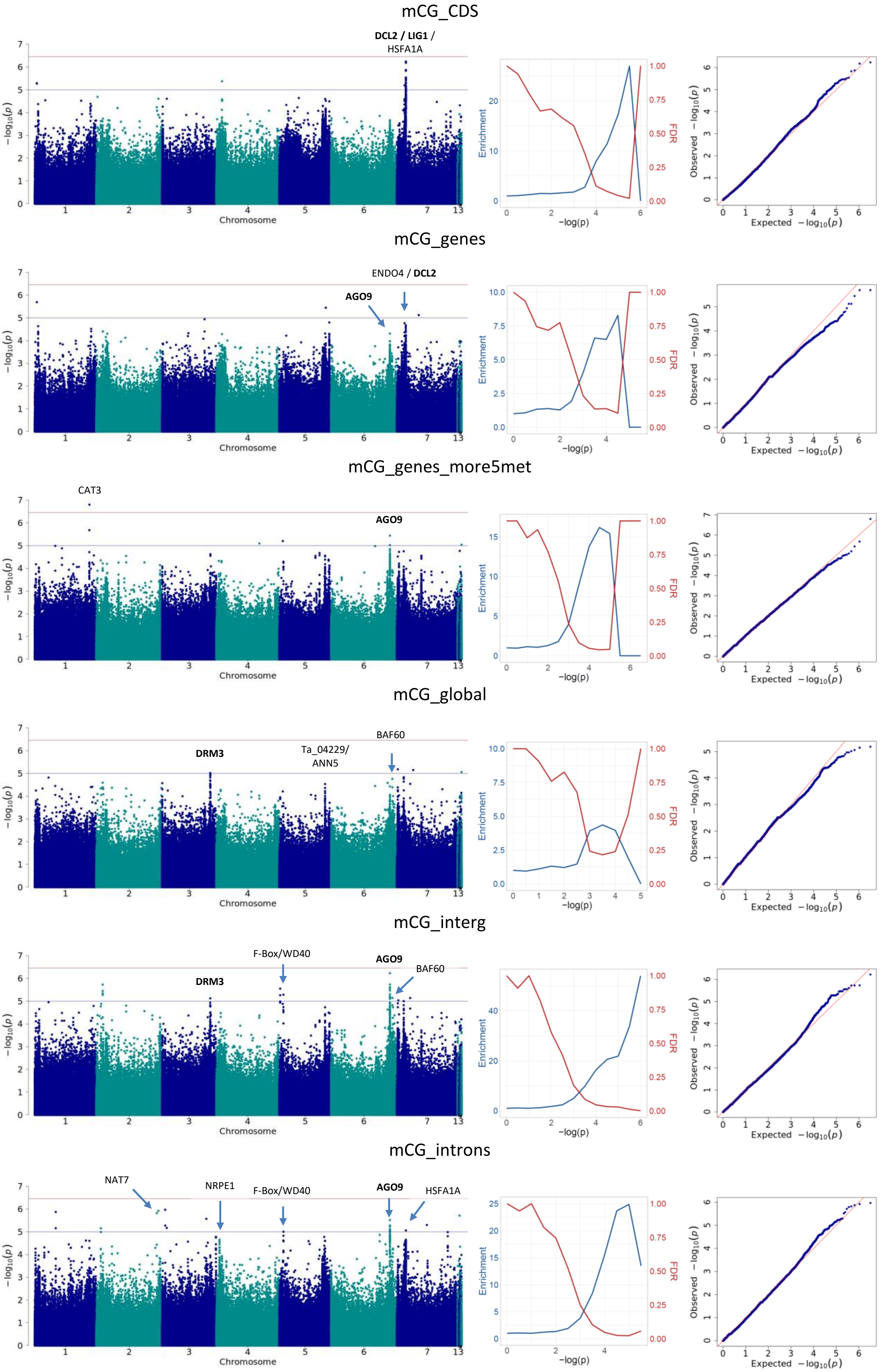
mCG_CDS
DCL2 / LIG1 / HSFA1A
mCG_genes
ENDO4 / DCL2
AGO9
mCG_genes_more5met
CAT3
AGO9
mCG_global
BAF60
DRM3
Ta_04229/ ANN5
mCG_interg
F-Box/WD40
AGO9
BAF60
DRM3
mCG_introns
NAT7
NRPE1
F-Box/WD40
AGO9
HSFA1A
Chromosome
Enrichment
FDR
-log(p)
Expected −log10(p)
Observed −log10(p)

mCG_prom

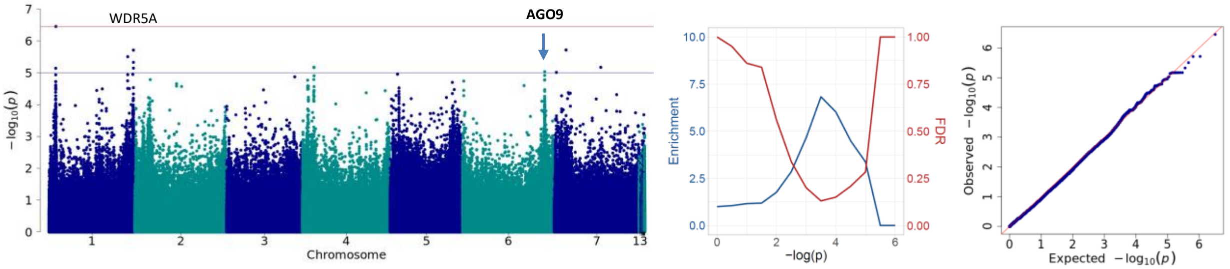

mCG_TEs

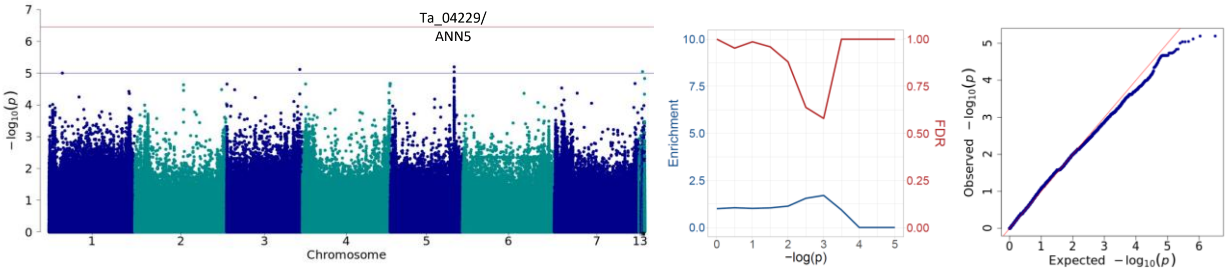

mCHG_CDS

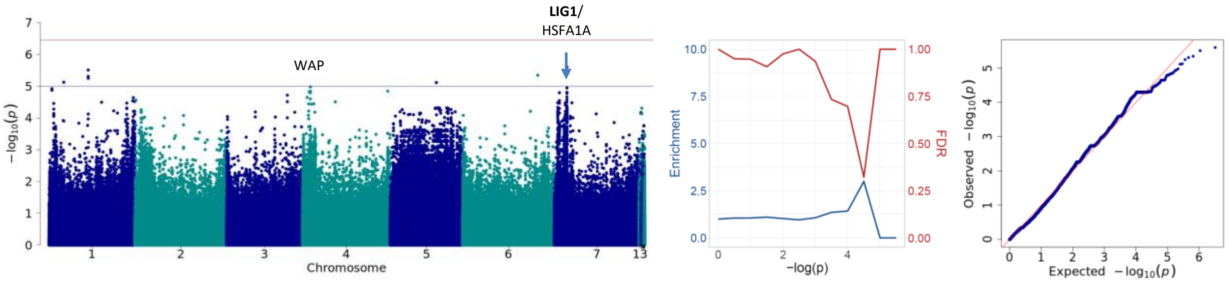

mCHG_genes

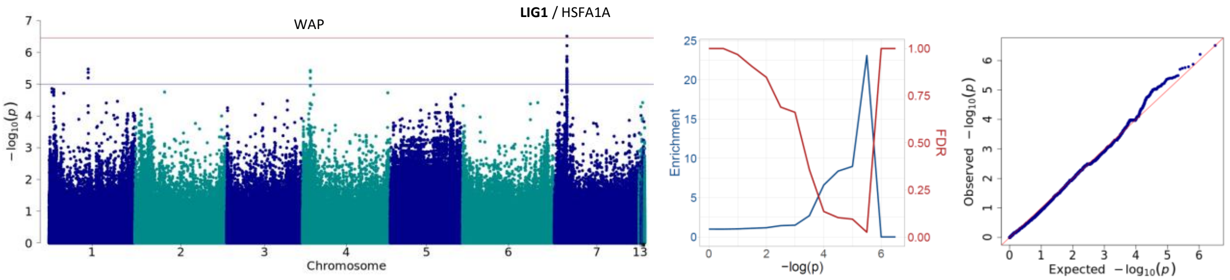

mCHG_genes_more5met

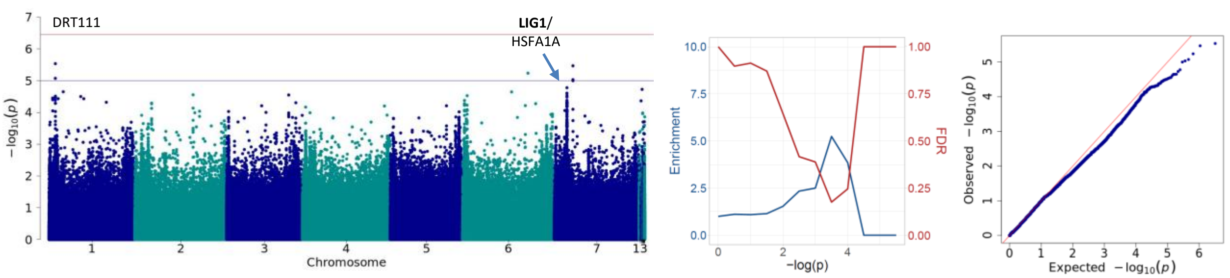

mCHG_global

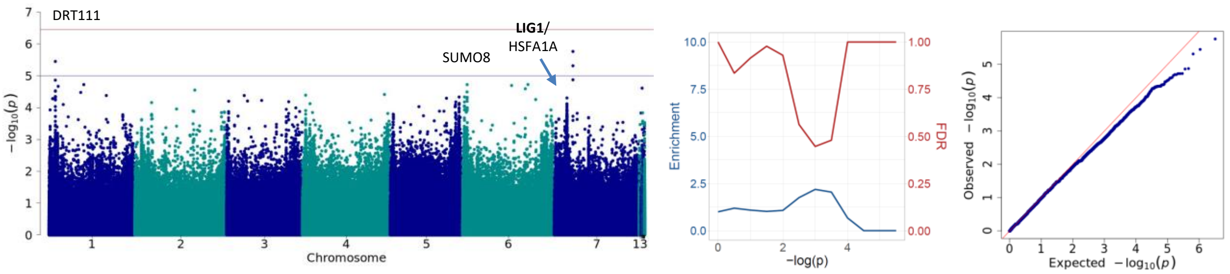

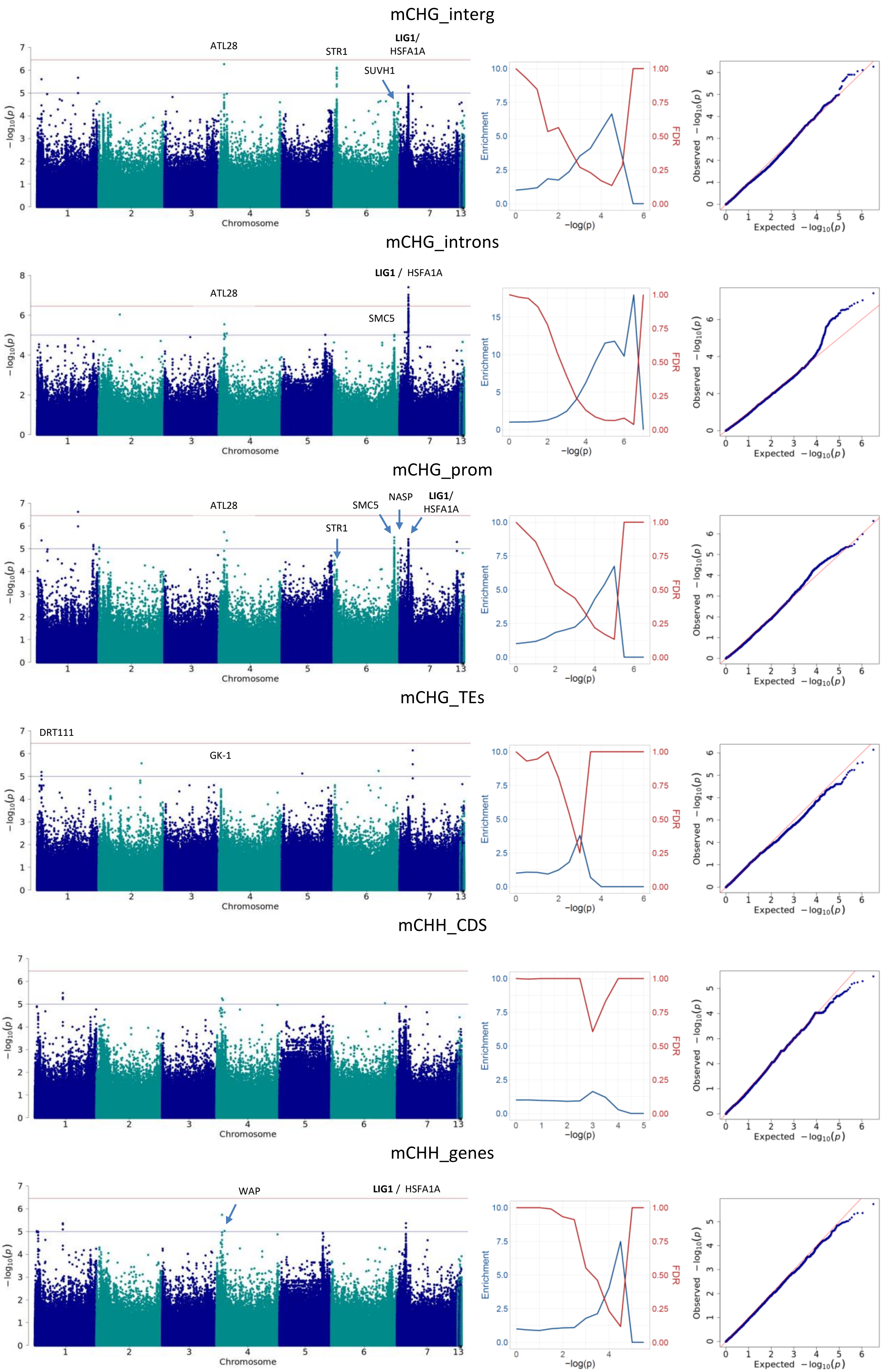
mCHG_interg
ATL28
STR1
LIG1/
HSFA1A
SUVH1
Chromosome
Enrichment
FDR
-log(p)
Expected
Observed
mCHG_introns
LIG1 / HSFA1A
ATL28
SMC5
mCHG_prom
ATL28
SMC5
NASP
LIG1/
HSFA1A
STR1
mCHG_TEs
DRT111
GK-1
mCHH_CDS
mCHH_genes
WAP
LIG1 / HSFA1A

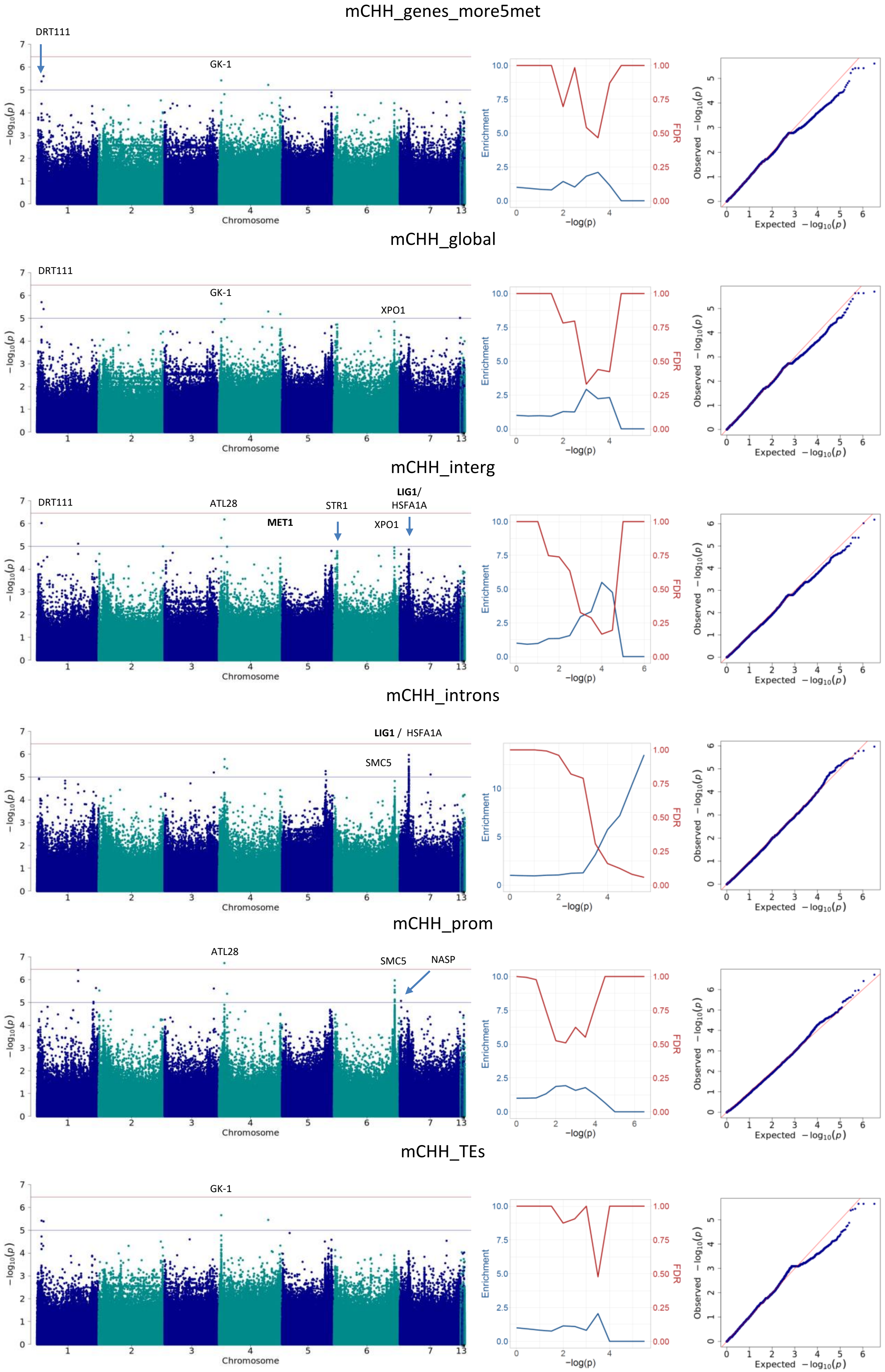

mCHH_genes_more5met
DRT111
GK-1
mCHH_global
DRT111
GK-1
XPO1
mCHH_interg
DRT111
ATL28
MET1
STR1
LIG1/
HSFA1A
XPO1
mCHH_introns
LIG1 / HSFA1A
SMC5
mCHH_prom
ATL28
SMC5
NASP
mCHH_TEs
GK-1
Chromosome
Enrichment
FDR
-log(p)
Expected
Observed
