# Supplementary material for: Genetic and environmental drivers of large-scale epigenetic variation in Thlaspi arvense
Source: PLoS Genet. 2022 Oct 12;18(10):e1010452. doi: 10.1371/journal.pgen.1010452 (PMC9591053; doi:10.1371/journal.pgen.1010452)
Supplement: S4 Table — Coverage correction indicates that, prior to GWA, residuals were extracted from a linear model with log(coverage) as predictor. INT indicates Inverse Normal Transformation. more5met: Mean methylation of genes with methylation > 5% across all lines. (PDF) [file pgen.1010452.s008.pdf]

S4 Table. List of all mean methylation variables used for GWA and climate correlation.

Trait	Outliers removed	Coverage Correction	Transformation applied
mCG_global	5	yes	none
mCHG_global	3	yes	none
mCHH_global	0	yes	none
CG_genes	7	yes	INT
CHG_genes	3	no	INT
CHH_genes	3	no	INT
mCG_CDSs	3	no	INT
mCHG_CDSs	3	no	INT
mCHH_CDSs	3	no	INT
mCG_introns	5	no	INT
mCHG_introns	3	no	INT
mCHH_introns	3	no	INT
CG_intergenic	5	yes	none
CHG_intergenic	0	yes	none
CHH_intergenic	0	yes	INT
mCG_TEs	2	no	none
mCHG_TEs	0	no	none
mCHH_TEs	0	no	none
mCG_prom	8	yes	none
mCHG_prom	3	yes	none
mCHH_prom	3	yes	none
CG_genes_more5met	5	yes	none
CHG_genes_more5met	0	yes	none
CHH_genes_more5met	0	yes	none

INT: Inverse Normal Transformation
