# Supplementary material for: Genetic and environmental drivers of large-scale epigenetic variation in Thlaspi arvense
Source: PLoS Genet. 2022 Oct 12;18(10):e1010452. doi: 10.1371/journal.pgen.1010452 (PMC9591053; doi:10.1371/journal.pgen.1010452)
Supplement: Attachment — Submitted filename: Response to reviewers.docx [file pgen.1010452.s012.docx]

MANUSCRIPT: Genetic and environmental drivers of large-scale epigenetic variation in *Thlaspi arvense*.

**Response to reviewers**

Thank you very much for allowing us to revise our manuscript! We found the comments and suggestions of the reviewers very useful and knowledgeable. They helped us to significantly improve our manuscript. Below we respond to each comment individually, with our responses in blue. Mentions of line numbers refer to the “marked-up” copy of the manuscript, they might of course differ in the clean revised copy.

Reviewer #1: Beyond Arabidopsis, profiling of natural diversity in the patterns of DNA methylation in large populations is scarce, and especially profiling using whole genome bisulfite sequencing. Thus, this manuscript is quite novel in that respect; the authors profile genome-wide patterns of DNA methylation in 207 pennycress lines, sampled from 36 natural populations. Both genetic background and climate of origin were found to be significantly associated with methylation variation.

This manuscript was a pleasure to read, thoughtful and polished.

Thank you for saying this!

Overall, the notable findings were quite similar to prior work in Arabidopsis. SNPs were found associated with variation in average methylation for different genomic features; and candidate genes were identified including known and novel candidate players. Average methylation levels show an association with climate of origin, colder climates are associated with reduced methylation, particularly CHH. It is interesting too that CG/CHG at TEs is negatively associated with the max temp/month climatic variable. Many DMRs also showed an association with both climate of origin and SNPs, with trans-SNPs being the strongest predictor. It would be great to have paired expression data, but that would be a substantial undertaking (not suggesting it).

These are definitely notable findings and great to see replicated beyond Arabidopsis. I guess one thing I wanted to discover is a strong argument to support why we do indeed need to do these studies in plants with larger, more TE rich genomes! I think we do, but do these results suggest that Arabidopsis really is a good enough model after all? Perhaps some more compare/contrast with the major conclusions from Arabidopsis work could be included in the discussion?

We thought that pointing out the non-representativeness of the *A. thaliana* genome would be sufficient to understand the value of studying epigenetic variation also in other species. However, after this comment we realized we should be more specific. We have now added a few more sentences to the Introduction (lines 123-131) that stress the difficulty to generalize, and that species with larger genomes could be different, e.g. because more TEs = more chances for epimutation and transposition = potentially generally more dynamic genomes, with greater relative importance of epigenetic processes.

In the Discussion there are already several instances where we compare our results to those from *A. thaliana*. Sometimes they agree, sometimes they don’t. We did not think another systematic comparison of Arabidopsis vs. Thlaspi was necessary.

Otherwise, I have just a couple of minor suggestions that should be addressed:

DRM3:
DRM3 is identified as a candidate - it has been reported previously that members of the DRM3 clade in plants lack conserved residues important for cytosine methyltransferase activity (https://doi.org/10.1371/journal.pgen.1001182 and doi: 10.1186/1756-0500-7-721); and hence while DRM3 affects methylation levels, it is likely a catalytically inactive paralog that promotes DRM2 activity. Based on the genome sequence, would the pennycress DRM3 be predicted to be active on inactive?

Thank you for this useful remark! There are 5 orthologs of *AtDRM3* in the *Thlaspi arvense* genome, but the gene where we observed the peak is the most similar one (76.72 % identity, see Fig below). After multiple sequence alignment, we found that this specific ortholog also lacks the conserved residues, particularly the proline-cysteine motif that binds to cytosine during catalysis. We have added this information to the text (line 239) and in Fig. 3.

Fig.: Multiple sequence alignment of AtDRM2, AtDMR3 proteins and corresponding T. arvense orthologues predicted proteins. The T. arvense orthologue of AtDRM3, where we observed the GWA peak is highlighted in yellow.

SNP-DMR associations:
Genetic variance in trans explained the largest proportion of DMRs (line 295), wondering if this is associated with many different SNPs or are there a few large effect SNPs (‘hotspots’) associated with many DMRs (eg AGO9, DRM3 or even CMT?)? Perhaps a chromosomal dot plot for associations of DMR vs SNP along the chromosomes could help visualise this (eg see Fig 3 in Dubin et al 2015 https://doi.org/10.7554/eLife.05255.007)? If there are some large effect SNPs/genes, should these be added to the model, especially if temperature also affects average methylation – could there be an environment x major-effect-SNP interaction?

These are interesting questions, and of course we also thought about GWA of individual DMRs, but in the end we decided against it, because this would have required to re-analyse with different software, but also because we knew from a preliminary GWA of a small subset of DMRs, that this would become rather difficult and error-prone.

First, many individual DMRs will often be very strongly associated with population structure and therefore inevitably also with many SNPs. This is strongly exacerbated by the distribution of DMRs methylation, which differently than average methylation is often, but not always, bimodal. Thus there will be many false associations - a problem we don’t have in GWA of average methylation where the population structure correction we apply is sufficient. Other GWA software than the one we used could be more suitable to tackle these problems, but this would require a re-analysis of our data, and given the number of DMRs in our study this would be computationally very demanding and time-consuming. Moreover, in our GWAs of average methylation we already found that even the strongest SNPs have only moderate effect sizes, so we do not think adding any of these to the model would influence the results much. Finally, to answer the first question, DMRs strongly associated to trans genetic variance in the variance decomposition analysis are almost surely associated to many SNPs and not to few. This is because they are strongly associated to an IBS matrix made with all SNPs and therefore to population structure (SNP-based heritability). A strong association with a single SNP would unlikely result in a strong association with trans genetic distance. In summary, a GWA of individual DMRs would have been long and complicated, and very likely accompanied by many statistical challenges. We therefore preferred not to look for SNPs underlying individual DMRs. We were hoping that our paper would also be substantial and interesting enough without such analyses.

Minor:
- it could be useful to label the major geographic collection locations in Fig 1a for non-European readers?

We improved Fig 1A by using a finer grid for the Copernicus satellite data, which makes coastlines and the whole figure much more neat and precise. Nevertheless we prefer not to label the geographic regions, to keep the figure simple, and because the regions play no critical role in our analyses. What matters is that there are clusters (= geographic structure that we take into account in our analyses), and that the sampling covered a large latitudinal gradient. Both is clear from the figure as it is. We trust that all of our readers will recognize Europe. Lastly, we also tried adding country borders, but this renders badly on coastlines, overlapping imprecisely with the gridded satellite data, so we prefer to only show the latter.

- line 148 it would be good to also include a table (or the state the average) conversion rate and mapping rate.

We have included the number of mapped reads, coverage and non-conversion rates in Table S2.

- line 257 are the bioclimatic data for each location available somewhere? I couldn’t see them but these would be required to replicate the findings; can a supp table be added.

We now provide the bioclimatic data for our studied populations in Table S7. Still, the climate data are open access from the Copernicus programme (doi.org/10.24381/cds.151d3ec6) and the locations of our populations were already in Table S1, so it is also possible to independently replicate our analyses.

- line 288 – the DMR list and/or co-ordinates might also be provided eg on Zendo.

They were already on Zenodo, but we forgot to mention this in the “Germplasm, data and code availability” statement. We added this now, together with the Variance Decomposition results.

- line 467 please provide a specific reference or link to the ‘reference genome’; what was the reference used?

We added the missing reference.

Reviewer #2: The manuscript by Galanti and colleagues describes analysis of factors underlying methylation variation in 207 lines of pennycress. Genetic variation is the main driver of global methylation variation, however climate of origin also has a small impact. The results largely recapitulate what has been demonstrated previously in Arabidopsis, making the manuscript of narrower interest. Generally, the analysis is sound and the conclusions justified, however I have a few suggestions for improvement.

The reviewer is correct that our findings recapitulate some of the observations already made in *A. thaliana*. However, and this point was also explicitly raised by Reviewer 1, this was not necessarily to be expected, given that *A. thaliana* is an outlier when it comes to genomic composition, including the extent of DNA methylation and its frequency in different genomic contexts. We believe that showing that some general patterns and trends hold also in a species with a considerably larger and TE-richer genome has merit on its own. Generalization is an important goal in science, and it is necessarily required to expand the research to a broader range of species.

See also our response to a related comment by Reviewer 1 above.

Major points:

Lines 79-81: The statement that CHH methylation is maintained by persistent de novo methylation by RdDM is only true in euchromatin. Throughout heterochromatin, CHH methylation is maintained by CMT2 in a mechanism similar to maintenance of CHG methylation. It is preferable to separate these two types of methylation during analysis. For example, changes in CMT2 alleles impact CHH methylation in heterochromatin but not in euchromatin (see Sasaki et al 2019 PLoS Genetics). I recognize that heterochromatin might not be clearly defined in pennycress, but perhaps proximity to genes could be used as a proxy, or ChIP-seq analysis of histone modifications could be completed in a reference line.

This is a very good point and we now state the role of *CMT2* more explicitly in the introduction (line 80). However, previous Arabidopsis studies (Kawakatsu et al. 2016, Sasaki et al. 2019) used not only information about eu- vs. heterochromatin, but also data from knockout lines, which allowed to identify TE families that are controlled by *DRM2* (RdDM) vs. *CMT2*. Such mutants and data are currently not available in Thlaspi. We therefore preferred to keep our analysis simpler, using only available annotated features, instead of somewhat arbitrarily defining euchromatin and heterochromatin.

The abstract mentions 207 lines, but really it is 36 populations that were studied. The smaller number of independent populations might explain the limited statistical power of the GWAS.

Of course this population structure affects statistical power, and we had already mentioned that our nested design might decrease statistical power (line 398). However, we should emphasize that the genetic (and epigenetic) variation within populations is oftentimes higher than that between populations, as evident e.g. from the PCA based on genetic variants in Fig. 1. Only few populations cluster tightly together, while many individual lines are genetically (and epigenetically) more similar to lines from other populations. Thus there is substantial variation among our 207 lines that can be used to e.g. associate genetic with epigenetic variation, as long as the non-independence created by the population structure is properly accounted for.

Clearly genetic variation is the main driver of global methylation variation. However, the manuscript really favors the hypothesis that climate of origin is an important factor. While Figure 5C is compelling that CHH methylation is more likely to be explained by environmental factors than other contexts of methylation, this is still minor compared to the effects of genetic variation. The manuscript would be improved by a more balanced approach to the results.

We state in many places (e.g. lines 34, 142-143, 324, 329, 451, ...) that genetic background is the strongest predictor. Of course this was our default expectation, based on the literature and our biological understanding. But it was also less surprising, whereas the association with climate, and in particular its differences between sequence contexts, we found more exciting, not only because some of us have a background in ecology, but also because such relationships have been less studied. Isn’t it normal to find more novel results also more exciting? We found methylation associations to climate impressive, particularly if one does not expect to find much a priori. The fact that genetics explains more variation, does not make environmental associations irrelevant. Advances in Science also come from further investigation of drivers with smaller effects. We double-checked our wording throughout the text, and we don’t think that we have anywhere overstepped the limits of what we could legitimately conclude.

It is surprising that the manuscript does not include discussion of Shen et al, PLoS Genet 2014, which demonstrated that alleles of CMT2 have been selected in variable climates (in Arabidopsis).

Thank you for this excellent suggestion! We have now included the paper in the discussion (lines 443-445).

There is no mention of how bisulfite conversion was assessed in these libraries. Given the higher-than-expected CHH methylation values, analysis of chloroplast sequences or lambda phase spike-ins is essential.

We have added bisulfite non-conversion rates and mapping statistics as a supplementary Table S2. However, even considering these non-conversion rates, weighted CHH methylation (12.3% average) is still higher than any other Brassicaceae and most analysed plant methylomes (Niederhuth et al. 2016).


Minor points:

I was unable to find an entry for PRJEB50950 in the ENA Sequence read archive.

This is because the data are searchable only after the release date (ena-docs.readthedocs.io/en/latest/faq/release.html). We will release the data immediately upon acceptance of our manuscript.

Line 110: Methyltransferase 1 is commonly abbreviated as MET1, or to be consistent with non-plant literature, DNMT1, not DMT1.

We fixed this everywhere, thanks.

Figure 3D says NRPB1L rather than NRPB10L

Thank you for spotting this mistake. We fixed it everywhere.

Reviewer #3: Galanti et al. provides a comparative analysis and GWAS of methylation across natural populations of Thlaspi arvense. Based on their analysis, genetic factors are the major driver of DNA methylation variation It also appears that this is a species that has potentially lost most of its gbM, however, I have major concerns about the robustness of the DNA methylation analysis that need to be addressed. It seems unlikely to me that these corrections could be completed in a short period.

1. I may have missed it, but I did not see any mapping statistics rates for any of the samples. For whole-genome bisulfite data, conversion or non-conversion rates are necessary. This is essential to evaluate the quality of the data.

Thank you for pointing this out. We now provide these stats in the supplementary Table S2.

2. It is not clear how the authors are calculating their methylation levels. Multiple approaches are used in the literature (see discussion in Schultz et al. https://www.cell.com/trends/genetics/fulltext/S0168-9525(12)00171-0). Some methods of calculation are strongly affected by factors such as read coverage. The preferred method is the “weighted methylation level” and allows for a more direct comparison between species. Also, by limiting their analysis to those cytosines with coverage in all samples, the author’s may also be missing the direct effects of genetic variation on methylation levels.

To calculate average methylation of multiple cytosines (regions or genomic features) we calculated the averages of individual positions (methylated/total reads) and then averaged across positions. This is ‘mean methylation’ sensu Schultz et al. 2013. Although it is true that this mean methylation was sometimes correlated to coverage, we corrected for it by using coverage-corrected residuals in the GWA (lines 596-599). Moreover, we did not limit our analyses to cytosines with coverage in all samples, but we included cytosines with coverage >3 in ≥75% of the samples (line 536-538), i.e. a much larger fraction of cytosines. For this, we employed methods for handling missing values.

Following the suggestion of the reviewer, we now additionally calculated weighted methylation (Schultz et al. 2013) and repeated all analyses. While climate associations and results of DMR variance decomposition were very similar, the results differed more for the GWA where some peaks close to a priori candidates, e.g. the one close to *AGO9*, were lost. As such peaks at functionally relevant genes had indicated to us that our analyses were (at least partly) meaningful and tracing true biological patterns, we thought about potential drawbacks of weighted methylation.

Two reasons why GWA results could differ between weighted and unweighted methylation data are that the weighted data are much more affected i) by non-random distribution of coverage along the genome, emphasizing aberrant coverage regions and ii) by structural and copy number variants, which are very abundant in Thlaspi (this is part of another ongoing study) because of its high TE content/mobility. Samples with the same structural or copy number variants may have high coverage of some particular genes or TE families (involved in this structural variant), and this may strongly impact weighted DNA methylation and increase the similarity of these samples. For example, samples with a high copy number of a specific TE family, will have high coverage of that family, which will have a high impact on the weighted methylation only in those samples. However this then reflects structural variation rather than some candidate genes affecting DNA methylation, and it will thus distort GWA results.

While the problem may be small in species with a simpler genome and few TEs, we suspect it might be problematic in Thlaspi, and outweigh the benefits of using weighted methylation. We therefore decided for a compromise, and we now present weighted methylation results (allowing for comparison with other species and studies) for general patterns (average methylation levels in different contexts, Figure 2), but unweighted methylation results where we associate methylation of individual samples with genomic or environmental variation. We explain this now also in the manuscript, and we clearly state which type of data is used where.

3. Line 185-196, Fig S2. I have a major concern regarding these results. It is highly unusual for genes with TE-like methylation (TeM) in greater abundance than CG-only gene-body methylation (gbM). While there are exceptions, such as Eutrema salsugineum, which have lost gbM, I suspect that the author’s results are due to inclusion of intronic/UTR methylation. This is supported by the GO-term analysis, which shows enrichment of houskeeping genes in TeM methylated patterns. In species like Eutrema, these genes are unM, while typically they are gbM in other species. From a straight-forward reading of the author’s methods, they are using DNA methylation within gene-bodies, including UTRs, CDS, and introns. Plant genes frequently have intronic TEs which contain extensive non-CG methylation. This is true even on CG-only gene-body methylated genes (gbM). If intronic cytosines are not excluded from such an analysis, this can lead to misinterpretation and misclassification of gbM genes as TE-like non-CG methylation. UTRs can also be problematic, as inaccurate UTR annotation in many species can also lead to over-representation of non-CG methylation in genes (see Niederhuth et al. https://genomebiology.biomedcentral.com/articles/10.1186/s13059-016-1059-0). I would also recommend a more rigorous approach for classification, such as those based on a binomial test of methylation in each gene, originally described by Takuno & Gaut (see <https://academic.oup.com/mbe/article/29/1/219/1749122?login=true>).

Following this comment, we completely reanalysed our data limiting the calculation of gene body methylation to coding sequences (CDS) and using a binomial model (Takuno and Gaut) to test for their methylation, borrowing also from Niederhuth et al. 2016 (see lines 564-579 in the Methods). Although the number of methylated CDS is now slightly lower than before (with whole genes and threshold-based methylation classification), the results remain qualitatively very similar. About 6-8% of genes are methylated in each sequence context (Table S3).

The number of gene-body-methylated (gbM) genes, i.e. methylated only in CG, remains very low in all lines, and it becomes even lower if one looks for consistent gbM across lines (only 81 genes methylated only in CG in ≥70% of the lines). Thus, this confirms that gbM is nearly absent in Thlaspi (Nunn et al. 2022), although some minor variation between lines exists both in the number (between 89 and 286) and composition of gbM genes.

When a CDS is methylated, it is usually methylated in all contexts, showing TE-like methylation signature, which is present in about 5% of the genes. Upon our re-analysis, these genes remain enriched for some housekeeping functions (eg. Nucleotide biosynthesis, protein catalysis, nuclease activity…). Since TEs are enriched in these genes compared to an average gene (8x more likely in a TE-like methylated gene), it looks like the strong TE expansion that occurred in *T. arvense* might have affected some housekeeping genes, which are usually gbM or unmethylated in other species, without compromising viability. Although a more accurate annotation of UTRs might decrease the number of these genes, the small changes obtained removing introns and all annotated UTRs suggest otherwise.

4. If the number of gbM genes is indeed so low, then the authors should check for the presence/absence CMT3 and other mutations.

The number of gbM genes is indeed very low, but so far we did not look into sequence details. *CMT3* is expressed in *Thlaspi arvense*. We added this to the discussion (lines 380-385). Additionally, we ran GWA using the number of gbM genes as phenotype, and we found no associations with any variants close to *CMT3*. Instead, we detected a clear and significant peak in a region without know methylation machinery genes (Fig S2).

**Have all data underlying the figures and results presented in the manuscript been provided?**
Large-scale datasets should be made available via a public repository as described in the *PLOS Genetics* [data availability policy](http://journals.plos.org/plosgenetics/s/data-availability), and numerical data that underlies graphs or summary statistics should be provided in spreadsheet form as supporting information.

Reviewer #1: **No:** line 257 - are the bioclimatic data for each location available somewhere?

We now provide the bioclimatic data for our studied populations in Table S7. We extracted these data from the Copernicus programme (doi.org/10.24381/cds.151d3ec6), which is open-access.

Reviewer #2: **No:** Methylation data should be available via the ENA sequence read archive, but I couldn't find them.

This is because the data are searchable only after the release date (<https://ena-docs.readthedocs.io/en/latest/faq/release.html>). We will release the data immediately upon acceptance of our manuscript.

Reviewer #3: Yes
