# Supplementary material for: Genetic and environmental drivers of large-scale epigenetic variation in Thlaspi arvense
Source: PLoS Genet. 2022 Oct 12;18(10):e1010452. doi: 10.1371/journal.pgen.1010452 (PMC9591053; doi:10.1371/journal.pgen.1010452)
Supplement: Attachment — Submitted filename: Response to reviewers 2.docx [file pgen.1010452.s013.docx]

MANUSCRIPT: Genetic and environmental drivers of large-scale epigenetic variation in *Thlaspi arvense*.

**Response to reviewers**

Reviewer #1: My concerns and suggestions have all been addressed, thank you.

Reviewer #2: The authors have addressed each of the points I raised, however now that I can see the bisulfite conversion rates (supplemental table 2), I am concerned that almost 10% of the samples have quite high non-conversion rates (>1%). I would have thrown out any sample that converted this poorly because it inflates CHH signals by a substantial margin. The authors argue that even considering the poor non-conversion rates, CHH methylation is high for Brassicaceae. I have no quibbles with that conclusion, given most of the samples look good. However, I do not have enough experience with population-level methylation analysis to know whether such a distortion might impact the findings of this paper. I am concerned because the poorly converted samples are not randomly distributed in the set. For examples, TA_SE_01 has an average non-conversion of 1.25%, with 5 of 6 libraries poorly converted, while TA_DE_06 has an average non-conversion of 0.17%. Perhaps the editor or another reviewer has more experience and can weigh in on this concern. Alternatively, perhaps the authors can confirm their findings with the poorer libraries removed from the analysis.

Reviewer #3: I thank the authors for addressing my comments and the extensive work they have done.

I only have one comment on this latest version. In looking at the non-conversion rates in table S2, it looks like the lines with a high non-conversion (>1%) cluster together in the table (rows 112, 122-129). Are these lines are related or collected from a similar region? Since the authors used mapping to the chloroplast genome to calculate the non-conversion rate, the higher non-conversion rate in these lines could be indicative of nuclear insertion of chloroplast sequences. Such sequences are usually methylated. If not present in the genome assembly, these reads could map to the chloroplast and result in the perception of a higher non-conversion rate than there actually is. I do not in think that this will affect the results of the paper. The non-conversion rates are still low enough that they are unlikely to have much impact and if I am right that this is maybe an artifact of a nuclear chloroplast insertion, then the actual non-conversion is much lower. However, the authors should probably check whether these samples show any odd clustering that might affect their results.

We thank the reviewers again for such careful and constructive evaluation of our work! We carefully investigated the reasons for the high non-conversion rates (NCRs) and thought about different options for resolving the problem.

Starting from Reviewer #3, we can quite confidently exclude that the high non conversion rates are caused by Nuclear Insertions of Plastid DNA (NUPTs). These insertions are indeed present in Thlaspi, and they did influence non-conversion rates in initial calculations based on all sequences with high similarity to the chloroplast genome. In these calculations, some samples had NCRs up to 5-6%, but the problem was in a specific region (most likely corresponding to the NUPT), and we already solved this in the previous revision, by simply removing that region from our calculations.

We also thought about different methods of calculating NCRs, and we tried a few different ways, including using an algorithm implemented in METHimpute (https://bmcgenomics.biomedcentral.com/articles/10.1186/s12864-018-4641-x) and calculating average methylation of unmethylated CDS (there are many in Thlaspi). The results were generally very similar to those in Table S2, and we therefore believe our NCRs are correct, and the high values are likely due to some irregularities during library preparation.

Having established this, we asked which analyses might be affected, and how to best remove a possible bias. While we believe that sample clustering, general patterns of methylation and DMR calling and variance decomposition were not relevantly influenced by the problem, GWAS and correlation analyses with climatic variables might have been. The sample clustering did not show any patterns traceable to high NCRs, and general patterns of methylation were still based on 90% of samples with normal NCRs. DMRs were explicitly defined by having at least 20% difference in CG and 15% in CHG and CHH, so a 1-2% difference due to NCRs was very unlikely to influence the results. In contrast, some average methylation values, such as those of genes and CHH methylation in general, had low absolute values, with a small range of variation. In such cases, GWAS and climate correlations might be affected more relevantly by higher than usual NCRs. As our GWAs were already suffering from relatively low power, removing 10% from our 200 samples would have been critical and hidden many true associations with low and medium allele frequencies. However, the calculated NCRs, which are in fact the average methylation rates of non-methylated DNA, allowed us to quantify the average methylation overestimation. We therefore decided to apply the following correction for higher NCRs: we calculated, for each individual sample, the surplus non-conversion rate from a baseline of 0.6% (Fig 1), and subtracted this value from the mean methylation values. The baseline of 0.6% allowed us to correct only the ~20% of samples with highest non-conversion rates, leaving most of the data unaltered. We document this change now in the methods (lines 589-593 of the marked-up manuscript).

Figure 1: Non-conversion rates of all lines and the surplus non-conversion rates from a 0.6% baseline (orange). Only 39 samples (<20%) had non-conversion rate higher than 0.6% and surplus non-conversion higher than 0.

We would like to underline that such a correction would not be appropriate for correcting methylation of individual positions or regions, and is therefore uncommon. However, it is applicable to whole genomic feature classes such as genes or TEs, because these are based on millions of cytosines. This is confirmed by our GWAS results, which overall had much higher enrichment of a-priori candidates than before the correction, indicating that we are likely detecting more true positives. Moreover, after the correction we detected more non a-priori candidate genes with roles possibly affecting methylation (Table S6), including ESP3, a pre-mRNA-splicing factor ATP-dependent RNA helicase involved in post-transcriptional gene silencing, WDR5A, involved in histone H3-K4 methylation, NERD, a Zinc finger CCCH domain-containing protein involved in DNA methylation and few more.

In addition, and as a sanity check, we investigated whether the samples with high NCRs might still be driving the strongest GWAS association in Fig 3 of the manuscript. In Fig 2 below, we have highlighted the samples with high NCRs, by colouring them differently. One of the prior associations, between Scaffold_4:5850379 and intergenic CHH methylation, appeared dubious (Fig 2A below) and dropped indeed below the significance threshold after the correction (Fig 3 of the manuscript). However, all the other associations were clearly not driven by the high NCR samples (Fig 2B below). In the new Fig 3C of our manuscript, we therefore substituted the Scaffold_4:5850379 association for a more meaningful one, in Scaffold_1, close to DRT111, which is involved in DNA repair.

Figure 2: Plots corresponding to Fig 3C in the manuscript. Samples with high non-conversion rate (above 1) are depicted in red.

We made a few more changes to the GWAS results section in the manuscript, but the main message and the most important candidate genes remained unchanged. Finally, we also re-ran the association with climate variables and updated Fig 4 in the manuscript, but there was hardly any difference in the results.

In summary, while the higher NCRs in some of the samples are unfortunate and the concerns of the reviewers were understandable, we have not found evidence that these NCRs influenced the findings that we report in our manuscript. We hope that by accounting for high NCRs and by showing that this does not alter the outcome of our analyses, we have been able to convince the reviewers and the editor.

On a last note, upon correction, some phenotypes previously transformed with the Gaussianize function (R package LambertW), resulted closer to normality. Since this transformation can complicate reproducibility, due to different versions of the package and options, we did not transform these phenotypes and only applied Inverse Normal Transformation when necessary (lines 596-599 of the marked-up manuscript).
